# Supplementary material for: Basketball robot object detection and distance measurement based on ROS and IBN-YOLOv5s algorithms
Source: PLoS One. 2024 Nov 21;19(11):e0310494. doi: 10.1371/journal.pone.0310494 (PMC11581231; doi:10.1371/journal.pone.0310494)
Supplement: Attachment — Submitted filename: ReviewerComments.docx [file pone.0310494.s001.docx]

The authors sought to enhance the performance of basketball robots by advancing object detection capabilities. The study developed an advanced detection system that combines the robot operating system with a fusion algorithm integrating v5s and laser detection technologies. Notable enhancements included the incorporation of instance-batch normalization modules to improve the model's generalization. Although the study presents promising results, several recommendations are offered to improve the manuscript.

**Abstract**

-The abstract would benefit from a clearer articulation of the need for object detection in basketball robots. It should explicitly outline the motivation behind this research, including the specific challenges addressed and the significance of the study. Enhancing these aspects will provide a more comprehensive understanding of the research question and its relevance.

-Additionally, the manuscript should maintain a consistent written style throughout. For instance, the first percentage is presented as 0.95, while the second is given as 97.89%. It is important to standardize the format for presenting percentages. For example, the statement "The recall curve area and F1 value of the improved algorithm were 0.95 and 97.89%, respectively," should be revised to ensure uniformity in the presentation of numerical data.

**Introduction**

The following revisions are recommended for this section:

-Citation Requirement:

The sentence "In the Chinese Robot Competition, the basketball robot project has become an important event" requires a citation to substantiate the claim. Please include an appropriate reference to support this statement.

-Sentence Structure:

Some sentences should be divided into more digestible parts and revised for a more formal academic tone. For example, consider revising the following sentence:

"At present, traditional object detection algorithms such as binocular vision algorithms, deep convolutional neural networks, and support vector machines, although they exhibit high recognition accuracy, suffer from slow detection speeds and limited learning and generalization abilities [5]."

-Terminology:

Instead of using informal terms like "Part 1" and "Part 2," the manuscript should use more formal terminology such as "Section 1," "Section 2," etc., to enhance the academic quality of the document.

-Additionally, the contributions of the study should be highligted in this section.

**Related Work**

-Please ensure careful attention to typographical errors, such as the possible confusion between "ROC" and "ROS."

-Additionally, algorithms discussed in the literature should be systematically presented in a table. The table should include columns for features such as "Authors, Year," "Dataset," "Method," "Performance Metrics," and "Results." This will facilitate a clearer comparison and review of the proposed methods. Comparisons should be conducted using the same dataset to ensure consistency and validity. If a comparable public dataset is unavailable, it is essential to provide detailed information about the dataset used for each literature study, including its characteristics, in a designated column.

**Method**

-The method is described with clarity; however, a question arises regarding the choice of algorithm. Specifically, why did the authors select the YoloV5s architecture? Given that there are more recent versions of the Yolo algorithm, such as YoloV7 and YoloV8, it would be beneficial to explore whether the authors considered or combined Instance Batch Normalization (IBN) with these newer versions. A comparative analysis of the results from these subsequent versions would provide valuable insights and enhance the discussion.

-Minor correction required: In Figure 9, the color representing the proposed IBN-Yolov5s appears orange rather than yellow. Additionally, the color used for Yolov5s is intended to be purple, but this color is not discernible in the figure. Please adjust the color scheme to accurately reflect the intended distinctions.

-In Figure 10, the authors reference the YOLOv5-CBAM algorithm. However, this algorithm is introduced for the first time in Section 4.2. It would be beneficial to provide an earlier mention or detailed description of this algorithm within the manuscript to ensure clarity and coherence. Furthermore, there is no comparison of the IBN-YOLOv5s algorithm with the other algorithms discussed in the preceding sections; only YOLOv5-CBAM is compared. This discrepancy suggests a possible oversight. Please review and ensure that comparisons with all relevant algorithms are included and accurately represented.

**Discussion**

-A discussion section should be added to the article to provide a thorough analysis and interpretation of the results, contextualize the findings within the broader field, and address any implications or limitations of the study.
