# Supplementary material for: Basketball robot object detection and distance measurement based on ROS and IBN-YOLOv5s algorithms
Source: PLoS One. 2024 Nov 21;19(11):e0310494. doi: 10.1371/journal.pone.0310494 (PMC11581231; doi:10.1371/journal.pone.0310494)
Supplement: Attachment — Submitted filename: Reply to Reviewers Comments.doc [file pone.0310494.s002.doc]

**Reviewer #1:**

• There are a considerable number of typographical errors that need to be addressed.

Reply: Thank you very much for your feedback. Printing errors have been checked and corrected.

• The introduction doesn’t have to be in a single paragraph. I highly recommend dividing it into three paragraphs, as it is currently too long. One of these could focus on the research structure. Similar issues exist throughout the manuscript and should be addressed.

Reply: Thank you very much for your valuable feedback. The introduction section has been reorganized.

Presently, conventional object detection algorithms, including binocular vision algorithms, deep convolutional neural networks, support vector machines, and others, despite exhibiting high recognition accuracy, are characterized by a slow detection speed, poor learning abilities, and limited generalization capabilities [5]. In the context of basketball robot games, the object detection system equipped not only requires high detection accuracy and fast calculation, but also strong feature learning ability to cope with the impact of different lighting environments on the playing field. Therefore, based on robot operating systems, this study introduces the lightweight and high detection accuracy You Only Look Once version 5 small (YOLOv5s) algorithm as the object detection algorithm, and integrates an appropriate amount of instance-batch normalization network modules to improve the original algorithm and enhance its detection performance.

• In the related work section, there are some irrelevant studies that should be removed, and more relevant studies should be included.

Reply: Thank you very much for your suggestion. The related work has been replaced and supplemented.

Guo used an improved YOLOv5 model to detect various road surface diseases, optimized the YOLOv5 model, and introduced an attention mechanism to enhance the robustness of the model. The improved model was more suitable for deployment in embedded devices. The experimental results showed that compared with YOLOv5s and YOLOv4 models, the improved YOLOv5 model has increased mean Average Precision (mAP) by 4.3% and 25.8%, respectively. This method could provide technical reference for road disease detection robots [7].

The apple target recognition algorithm is one of the core technologies of apple picking robots. To automatically identify the apples that can be grasped and those that cannot be grasped in apple tree images, Bin proposed a lightweight apple target detection method for picking robots using an improved YOLOv5s. The experimental results showed that the proposed improved network model had recognition recall, accuracy, mAP, and F1 value of 91.48%, 83.83%, 86.75%, and 87.49%, respectively. The mAP of the improved YOLOv5 model increased by 5.05%, 14.95%, 4.74%, and 6.75%, respectively, and the model size was compressed by 9.29%, 94.6%, 94.8%, and 15.3% [10].

Targeted spray has always been one of the hot issues in the research field of plant protection robots. To solve the problem of precise spraying, Liu proposed a soybean phenotype information perception method based on improved YOOv5. The experimental results showed that the improved YOLOv5 had an mAP of 96.13%, FPS of 79, a 39% reduction in the number of model parameters, and a 55.56% reduction in weights. The correlation coefficient between the obtained data and the manual measurement of plant phenotype information was 0.98384, indicating higher accuracy and better consistency [14].

[7] Guo K, He C, Yang M, Wang S. A pavement distresses identification method optimized for YOLOv5s. Scientific Reports, 2022, 12(1): 1-15.

[10] Bin Y, Pan F, Lei X, Liu Z, Yang F. A real-time apple targets detection method for picking robot based on improved YOLOv5. Remote Sensing, 2021, 13(9): 1619-1620.

[14] Liu L, Liang J, Wang J, Hu P, Zheng Q. An improved YOLOv5-based approach to soybean phenotype information perception. Computers & Electrical Engineering, 2023, 106: 1-18.

• The description of the deployed training facility was not well-explained, especially regarding which GPU was used, which is crucial information.

Reply: Thank you very much for your feedback. The deployed training facilities have been supplemented with explanations.

In the experimental deployment, a desktop computer with 16 GB of RAM running on Windows 10 operating system was studied, and the CPU model was equipped with Intel (R) Core (TM) i5-9400F CPU. The GPU model was NVIDIA RTX 3080.

• It would be beneficial to specify which deep learning library was used.

Reply: Thank you very much for your valuable feedback. The deep learning library has been supplemented.

In the training of deep learning libraries, PyTorch was selected for research. It is easy to learn that it has a large number of high-quality pre-trained models and open source projects for reference and use, and has a wide range of applications in the field of object detection. It supports the implementation of various object detection algorithms, including the YOLO series. Its high-performance computing power can meet the real-time requirements of basketball robot games.

• A maximum iteration number of 150 seems quite low. Additionally, the batch size used during training should be specified.

Reply: Thank you very much for your feedback. The batch size and iteration times have been redefined.

The maximum number of iterations for the experimental setup was 300. The maximum number of iterations for model training was 400.

Figure 7 (b) shows the F1 value variation curve, and as the amount of iterations increased, the F1 values of all algorithms improved. When the number of iterations was 50, the F1 values of SSD, Mask RCNN, YOLOv5s, and IBN-YOLOv5s were 64.23%, 59.59%, 68.21%, and 71.56%, respectively. When the number of iterations was 150, the F1 values of SSD, Mask RCNN, YOLOv5s, and IBN-YOLOv5s were 82.43%, 71.09%, 80.97%, and 97.89%, respectively. The IBN-YOLOv5s model maintained the highest F1 value before and after iterations. When the amount of iterations exceeded 150, the F1 value changed gradually and stabilized at over 90%.

• The explanation of the model was insufficient and needs improvement.
Reply: Thank you very much for your valuable feedback. The explanation of the model has been improved.

With the combination of artificial intelligence and robotics technology, more and more professional robots are entering the public eye. Basketball robot competition, as a very good target system for autonomous robot research, is very suitable for conducting research on robot autonomous perception system object detection. However, traditional basketball robots have problems such as recognition difficulties, which seriously affect the recognition of robot targets and distance measurement based on recognition. To improve the performance of basketball robots in competitions, research was conducted to improve the object detection system. Firstly, a basketball robot object detection system based on robot operating system was designed. In the software layer of the object detection system, an algorithm that combines YOLOv5s and laser detection was used, and an appropriate instance batch normalization network module was introduced in the YOLOv5s algorithm to improve the model’s generalization ability.

• YOLO has many versions, for instance, YOLOv10, but the author used YOLOv5. It should be explained why YOLOv5 was selected, if possible.

Reply: Thank you very much for your suggestion. The reason for choosing YOLOv5 has been supplemented.

YOLOv5 is an algorithm that combines detection speed and accuracy in the field of object detection. While meeting the requirements of basketball robot detection accuracy, its lighter model is more suitable for application in basketball robot controllers with higher real-time requirements compared to other algorithms in the YOLO series. The YOLOv5 model abstracts object detection in images as a regression problem.

**Reviewer #2:**

The authors sought to enhance the performance of basketball robots by advancing object detection capabilities. The study developed an advanced detection system that combines the robot operating system with a fusion algorithm integrating v5s and laser detection technologies. Notable enhancements included the incorporation of instance-batch normalization modules to improve the model’s generalization. Although the study presents promising results, several recommendations are offered to improve the manuscript.

Abstract
-The abstract would benefit from a clearer articulation of the need for object detection in basketball robots. It should explicitly outline the motivation behind this research, including the specific challenges addressed and the significance of the study. Enhancing these aspects will provide a more comprehensive understanding of the research question and its relevance.

Reply: Thank you very much for your valuable feedback. The motivation behind this study, including the specific challenges addressed and the significance of the research, has been redefined.

With the combination of artificial intelligence and robotics technology, more and more professional robots are entering the public eye. Basketball robot competition, as a very good target system for autonomous robot research, is very suitable for conducting research on robot autonomous perception system object detection. However, traditional basketball robots have problems such as recognition difficulties, which seriously affect the recognition of robot targets and distance measurement based on recognition. To improve the performance of basketball robots in competitions, research was conducted to improve the object detection system. Firstly, a basketball robot object detection system based on robot operating system was designed. In the software layer of the object detection system, an algorithm that combines YOLOv5s and laser detection was used, and an appropriate instance batch normalization network module was introduced in the YOLOv5s algorithm to improve the model’s generalization ability.

The improved algorithm proposed in the study provides more reliable and rich information for the perception ability of basketball robots, as well as for their subsequent decision-making and action planning, thereby improving the overall technical level of the robots.

-Additionally, the manuscript should maintain a consistent written style throughout. For instance, the first percentage is presented as 0.95, while the second is given as 97.89%. It is important to standardize the format for presenting percentages. For example, the statement “The recall curve area and F1 value of the improved algorithm were 0.95 and 97.89%, respectively,” should be revised to ensure uniformity in the presentation of numerical data.

Reply: Thank you very much for your feedback. The writing style of the manuscript has been standardized.

The recall curve area and F1 value of the improved algorithm were 0.95 and 0.9789, respectively.
Introduction
The following revisions are recommended for this section:

-Citation Requirement:
The sentence “In the Chinese Robot Competition, the basketball robot project has become an important event” requires a citation to substantiate the claim. Please include an appropriate reference to support this statement.

Reply: Thank you very much for your valuable feedback. Appropriate reference citations have been provided.

In the Chinese Robot Competition, the basketball robot project has become an important event [2].

[2] Shi F, Hu X. Fuzzy dynamic obstacle avoidance algorithm for basketball robot based on multi-sensor data fusion technology. International Journal of Foundations of Computer Science, 2022, 33(7): 649-666.

-Sentence Structure:
Some sentences should be divided into more digestible parts and revised for a more formal academic tone. For example, consider revising the following sentence:
“At present, traditional object detection algorithms such as binocular vision algorithms, deep convolutional neural networks, and support vector machines, although they exhibit high recognition accuracy, suffer from slow detection speeds and limited learning and generalization abilities [5].”

Reply: Thank you very much for your valuable feedback. The sentence has been modified.

Presently, conventional object detection algorithms, including binocular vision algorithms, deep convolutional neural networks, support vector machines, and others, despite exhibiting high recognition accuracy, are characterized by a slow detection speed, poor learning abilities, and limited generalization capabilities [5].

-Terminology:
Instead of using informal terms like “Part 1” and “Part 2,” the manuscript should use more formal terminology such as “Section 1,” “Section 2,” etc., to enhance the academic quality of the document.

Reply: Thank you very much for your feedback. This section has been modified.

The research structure is divided into four parts. The section 1 is a review of relevant research. Section 2 is the design of a basketball robot object detection system based on Robot Operating System (ROS) and improved YOLOv5s. Section 3 is an experimental analysis of the performance of the object detection system. Section 4 is a research summary and shortcomings, and proposes future research directions.

-Additionally, the contributions of the study should be highligted in this section.
Reply: Thank you very much for your valuable feedback. The research contribution has been supplemented.

ROS, as a flexible robot software framework, can effectively manage and coordinate robot hardware and software components, thereby improving the overall performance and efficiency of robots. As an improved object detection algorithm, the research algorithm enhances the generalization ability of the model by introducing the Instance and Batch Normalization Networking (IBN-Net) module, and improves the detection accuracy when there is a large difference in the distribution between the test set and the training set. This is crucial for robots to adapt to object detection tasks in different environments and conditions.

Related Work
-Please ensure careful attention to typographical errors, such as the possible confusion between “ROC” and “ROS.”

Reply: Thank you very much for your feedback. The formatting error has been corrected.

ROS is currently the most widely used robot software development platform, which has been extensively studied by scholars due to its rich toolset, distributed design, and excellent scalability.

-Additionally, algorithms discussed in the literature should be systematically presented in a table. The table should include columns for features such as “Authors, Year,” “Dataset,” “Method,” “Performance Metrics,” and “Results.” This will facilitate a clearer comparison and review of the proposed methods. Comparisons should be conducted using the same dataset to ensure consistency and validity. If a comparable public dataset is unavailable, it is essential to provide detailed information about the dataset used for each literature study, including its characteristics, in a designated column.

Reply: Thank you very much for your valuable feedback. The relevant work summary table has been supplemented.

**Table 1. Summary Table of Related Work**

| Author | Time | Data set | Method | Performance indicators | Result |
| --- | --- | --- | --- | --- | --- |
| Tosun et al. | 2023 | Dynamic and static environments | Combination of Hybrid Algorithm and ROS | Planning efficiency, time efficiency | Has a significant impact on both planning and timing |
| Guo | 2022 | Road surface disease image | Improved YOLOv5 model with introduction of attention mechanism | mAP | Compared to YOLOv5s and YOLOv4, mAP increased by 4.3% and 25.8%, respectively |
| Singh et al. | 2021 | Simulate map environment | Building dynamic models using key and graph methods, ROS navigation model | Success rate of obstacle avoidance | Effectively achieving safe driving in simulation testing |
| Oliveira et al. | 2022 | Driver and passenger behavior data | ROS system vehicle data collection and analysis model, combined with facial detection algorithm | Abnormal behavior monitoring capability | Track abnormal situations by monitoring driver and passenger behavior |
| Bin et al. | 2021 | Apple Tree Image | Improved YOLOv5s Lightweight Apple Object Detection Method | Identify recall, accuracy, mAP, F1 value | The recall rate is 91.48% and the accuracy rate is 83.83%, mAP 86.75%, F1 value 87.49% |
| Zhang et al. | 2023 | Silicon image of wheat straw epidermis | Automatic detection method based on YOLOv5s, adding reverse Resunit and attention mechanism | Accuracy rate | The accuracy rate is as high as 98.88% |
| Liu et al. | 2023 | Chili image | Improved YOLOv5s combined with bidirectional feature pyramid network | Robustness | Good robustness in scenarios of day, night, and tree cover |
| Qian et al. | 2023 | Photovoltaic module image | The method of segmentation before detection, using an improved semantic segmentation model and YOLOv5s | Average IoU, Average Accuracy | Semantic segmentation model IoU 97.8%, accuracy 89.6% |
| Liu et al. | 2023 | Soybean phenotype information image | A soybean phenotype information perception method based on improved YOLOv5 | mAP, FPS number and weight of model parameters | mAP 96.13%, FPS 79, 39% reduction in model parameters and 55.56% reduction in weights |

Method
-The method is described with clarity; however, a question arises regarding the choice of algorithm. Specifically, why did the authors select the YoloV5s architecture? Given that there are more recent versions of the Yolo algorithm, such as YoloV7 and YoloV8, it would be beneficial to explore whether the authors considered or combined Instance Batch Normalization (IBN) with these newer versions. A comparative analysis of the results from these subsequent versions would provide valuable insights and enhance the discussion.

Reply: Thank you very much for your feedback. The reason for choosing YOLOv5 has been supplemented.

YOLOv5 is an algorithm that combines detection speed and accuracy in the field of object detection. While meeting the requirements of basketball robot detection accuracy, its lighter model is more suitable for application in basketball robot controllers with higher real-time requirements compared to other algorithms in the YOLO series. The YOLOv5 model abstracts object detection in images as a regression problem.

-Minor correction required: In Figure 9, the color representing the proposed IBN-Yolov5s appears orange rather than yellow. Additionally, the color used for Yolov5s is intended to be purple, but this color is not discernible in the figure. Please adjust the color scheme to accurately reflect the intended distinctions.

Reply: Thank you very much for your valuable feedback. The color issue in Figure 9 has been corrected.

**Figure 9 Running time of four algorithms**

-In Figure 10, the authors reference the YOLOv5-CBAM algorithm. However, this algorithm is introduced for the first time in Section 4.2. It would be beneficial to provide an earlier mention or detailed description of this algorithm within the manuscript to ensure clarity and coherence. Furthermore, there is no comparison of the IBN-YOLOv5s algorithm with the other algorithms discussed in the preceding sections; only YOLOv5-CBAM is compared. This discrepancy suggests a possible oversight. Please review and ensure that comparisons with all relevant algorithms are included and accurately represented.

Reply: Thank you very much for your feedback. The YOLOv5 CBAM algorithm has been described in detail, and the IBN-YOLOv5s algorithm has been supplemented and compared.

On the basis of verifying the precision, IoU, and time consumption of the IBN-YOLOv5s algorithm in the pre-order, the study would further test the practical application performance of basketball robots fused with IBN-YOLOv5s. Firstly, the comparison outcomes of the mAP of the five algorithms are shown in Figure 10. YOLOv5s-CBAN is an object detection algorithm that combines attention mechanisms (Channel Attention, Bottleneck Attention Network) to help the model focus more on important information during feature extraction, thereby improving detection performance.

**Figure 10 MAP results of five algorithms**

When the number of iterations was 40, the mAP values of the five algorithms, SSD, Mask RCNN, YOLOv5s, YOLOv5-CBAN, and IBN-YOLOv5s, were 78.23%, 65.01%, 82.16%, 77.21%, and 93.93%, respectively. When the number of iterations was 120, the mAP values of SSD, Mask RCNN, YOLOv5s, YOLOv5-CBAN, and IBN-YOLOv5s algorithms were 79.58%, 82.06%, 86.37%, 84.01%, and 98.43%, respectively.

Discussion
-A discussion section should be added to the article to provide a thorough analysis and interpretation of the results, contextualize the findings within the broader field, and address any implications or limitations of the study.

Reply: Thank you very much for your valuable feedback. The discussion section has been supplemented.

**4.3 Discussion**

To verify the feasibility of the improved IBN-YOLOv5s algorithm in basketball robot object detection, in performance testing, the F1 values of IBN-YOLOv5s were 97.89% after 150 iterations. The IBN-YOLOv5s model maintained the highest F1 value before and after iterations. When the number of iterations exceeded 150, the F1 value changed gradually and stabilized at over 90%. The reference [10] showed that YOLOv5 had a recognition recall rate of 91.48% and an accuracy rate of 83.83%, mAP 86.75%, F1 value 87.49%. It can be seen that the research algorithm has good performance. In the IoU ratio of processed images, as the number of training sets increased, the IoU values of processed images showed an increasing trend. When the training set size was 800, the IoU values of IBN-YOLOv5s were 0.96. Reference [13] showed that the detection model had an IoU of 97.8% and an accuracy of 89.6%, indicating the advantage of the proposed method in terms of IoU ratio. As the size of the training set increased, the signal-to-noise ratio of the image processed by the algorithm showed an increasing trend. When the size of the training set was 800, the SNR value of IBN-YOLOv5s was 0.98. The improved YOLOv5s recognition proposed in reference [11] had a recall rate of 91.48% and an accuracy rate of 83.83%, mAP 86.75%, F1 value 87.49%. It can be seen that the proposed IBN-YOLOv5s algorithm has better image processing performance compared to other algorithms. In the testing of the basketball robot system integrating IBN-YOLOv5s, the mAP value of the IBN-YOLOv5s algorithm was 98.43% when the number of iterations was 120. The improved algorithm proposed in reference [7] improved the mAP of the YOLOv5 model by 4.3% and 25.8% compared to YOLOv5s and YOLOv4, respectively. It can be seen that the algorithm has better accuracy and precision compared to other algorithms. The results indicate that the IBN-YOLOv5s model proposed by the research has robust performance and can effectively recognize different target objects in basketball robot competitions. Research is not only an exploration of the application of YOLOv5s algorithm in basketball robot games, but also the discovery of new technological paths and solutions through continuous optimization and improvement of the algorithm, promoting the technological innovation and development of deep learning technology in the field of sports robots, which is of great significance for promoting the progress of the entire sports industry.

**5. Conclusion**

The comprehensive performance of the improved algorithm raised in this study was excellent, but the IBN-YOLOv5s used in the study was a fixed parameter network structure, which will reduce the adaptability of the model. In future research, dynamic parameters can be selected to improve the structure of the network model for further testing.
